# Melatonin Use for Sleep in Assisted Living

**DOI:** 10.1093/geroni/igaa057.2354

**Published:** 2020-12-16

**Authors:** Punita Peketi, Sheryl Zimmerman, Stephanie Miller, Christopher Wretman, John Preisser, Philip Sloane

**Affiliations:** 1 Northeast Ohio Medical University, Twinsburg, Ohio, United States; 2 University of North Carolina at Chapel Hill, Chapel Hill, North Carolina, United States; 3 Cecil G. Sheps Center for Health Services Research, Chapel Hill, North Carolina, United States

## Abstract

Sleep problems are common among residents of assisted living (AL) communities, and in other settings, melatonin is used to promote sleep. However, melatonin use in AL is unknown; because it may have side-effects, this knowledge gap is concerning. To address this question, data were collected across 250 AL communities in seven states; analyses used weights whereby data were scaled to represent the entirety of the states. The majority of communities prescribed melatonin (82%), albeit to a minority of residents (9%). Prescribing was more common for those with anxiety, sleep-wake disorders, dementia, and various behaviors, in communities that had more staff and more favorable non-pharmacological attitudes (p<.05). Dosages varied from 0-45 mg and co-prescribing was common. This study is the first to examine melatonin prescribing in AL; use may be appropriate if, for example, it is a replacement for hypnotics. The variation suggests practices may be modifiable; further research is needed.

